# Antimicrobial Activity of *Antrodia camphorata* Extracts against Oral Bacteria

**DOI:** 10.1371/journal.pone.0105286

**Published:** 2014-08-21

**Authors:** Hsiu-Man Lien, Chin-Jui Tseng, Chao-Lu Huang, Yu-Ting Lin, Chia-Chang Chen, Ya-Yun Lai

**Affiliations:** 1 Department of Chemistry, Tunghai University, Taichung, Taiwan; 2 Department of Chemistry, National Chung Hsing University, Taichung, Taiwan; 3 Department of Life Science, National Chung Hsing University, Taichung, Taiwan; 4 Department of Food Science, National Ilan University, Ilan, Taiwan; 5 School of Management Department, Feng Chia University, Taichung, Taiwan; 6 Department of Applied Cosmetology, National Tainan Institute of Nursing, Tainan, Taiwan; 7 Yusheng Biotechnology Co. Ltd., Taichung, Taiwan; Taipei Medical University, Taiwan

## Abstract

*Antrodia camphorata* (*A. camphorata*) is a unique, endemic and extremely rare mushroom species native to Taiwan, and both crude extracts of and purified chemical compounds from *A. camphorata* have been reported to have a variety of significant beneficial effects, such as anti-tumor and anti-inflammatory activity. However, reports on the effects of *A. camphorata* against dental pathogens have been limited. Oral health is now recognized as important for overall general health, including conditions such as dental caries, periodontal disease and rheumatoid arthritis. *Streptococcus mutans* (*S. mutans*) and *Porphyromonas gingivalis* (*P. gingivalis*) are the most common bacteria associated with dental plaque and periodontopathic diseases, respectively. Thus, our study examined the ability of five various crude extracts of *A. camphorata* to inhibit the growth of dental bacteria and anti-adherence *in vitro*. Among the extracts, the ethanol, ethyl acetate and chloroform extracts exhibited the lowest MICs against *P. gingivalis* and *S. mutans* (MIC = 4∼16 µg/mL). The MIC of the aqueous extract was greater than 2048 µg/mL against both *P. gingivalis* and *S. mutans. In vitro* adherence of *S. mutans* was significantly inhibited by the addition of either the ethyl acetate extract or chloroform extract (MIC = 16∼24 µg/mL), while the ethanol extract (MIC = 32∼64 µg/mL) exhibited moderate inhibitory activity. Based on the result of this study, the ethyl acetate and chloroform extracts of *A. camphorata* may be good candidates for oral hygiene agents to control dental caries and periodontopathic conditions.

## Introduction

Oral health is now recognized as important for overall general health. Oral diseases such as dental caries, which is the most common chronic childhood disease, and adult periodontal infections are prevalent conditions. Maintaining good oral health has the potential to improve lifelong health. Dental caries, gingivitis, and periodontal infection are infectious diseases of multiple origins that can worsen and remit over time. *Streptococcus mutans* (*S. mutans*) is closely associated with the development of human dental caries and the most common pathogen isolated from human dental plaque [1]–[2]. *S. mutans* is transmitted primarily from mother to child by contact with saliva and high levels of *S. mutans* in the mother are the greatest indicator of caries risk among her children. Acquisition of *S. mutans*, coupled with high dietary sugar intake, establishes a favorable environment for caries development. Thus poor oral health, poor oral hygiene behaviors, and dietary patterns that promote high colonization with *S. mutans* may increase both the contamination of oral cavity and presence of substrates for bacterial growth.

Periodontitis is an inflammatory disorder of the periodontium that can eventually lead to tooth loss and the loss of supporting structures. *Porphyromonas gingivalis* (*P. gingivalis*), is present under both healthy and diseased conditions and has been proposed as a periodontal pathogen and etiological link connecting with periodontal disease [3]. *P. gingivalis* is a gram-negative oral anaerobe that expresses peptidyl arginine deiminase (PADI), which converts arginine to citrulline in normal tissues [4]. Naturally occurring herbal antimicrobial agents [5] have emerged as alternatives to oral antimicrobials, such as chlorhexidine [6]. In general, these herbal antimicrobial agents exhibit diverse structures that reduce the potential for the development of microbial resistance. The purpose of the present study was to evaluate the antimicrobial activity of the Taiwanese traditional herbal medicine Antrodia *camphorata* (*A. camphorata*), a unique, endemic and precious native species of mushroom. Taiwanese aborigines used *A. camphorata* to treat liver disease and prevent food and drug intoxication. Both crude extracts and purified chemical compounds of *A. camphorata* have been shown to have a variety of significant beneficial effects, such as anti-tumor [7]–[9], anti-inflammatory [10]–[12], anti-oxidant [13]–[14], and immuno-modulatory activity [15]–[16]. However, reports of inhibitory effects of *A. camphorata* against pathogens such as dental bacteria have been very limited. Therefore, the aims of this study were to evaluate different crude solvent extracts of *A. camphorata* against oral bacteria, including *P. gingivalis* and *S. mutans*.

## Materials and Methods

### 1.1 Materials

Chlorhexidine (CHX), which primarily affects gram-positive oropharyngeal microorganisms, was purchased from Sigma-Aldrich Co. Ltd (Chemie GmbH at Riedstrasse 2, Steinheim). *A. camphorata* was obtained from Yusheng Biotechnology Co. Ltd., Taichung, Taiwan. Three in-house working standards (AC-1: 4,7-dimethoxy-5-methyl-l,3-benzodioxole; AC-2: dehydrosulphurenic acid; AC-3: dehydroeburicoic acid) from *A. camphorata* for quantitative study and antibacterial assays were isolated, purified and determined by nuclear magnetic resonance (NMR) spectroscopy, Mass spectroscopy and elemental analysis. Reference oral bacterial strains *P. gingivalis* BCRC 14417 (ATCC 33277), *S. mutans* BCRC 15256 (ATCC 31383) and human gingival fibroblast (HGF) cells were purchased from Food Industry Research and Development Institute (Hsinchu, Taiwan).

### 1.2 Extracts of *A*. *camphorata*

Dried A. camphorata was used to prepare extracts in water, 50% ethanol, 95% ethanol, ethyl acetate and chloroform as the extraction solvents. A total of 200 mL of extraction solvent was added to 20 g of ground (passed through a 30 mesh screen) *A. camphorata*, followed by stirring (125 rpm) for 48 hours at room temperature. The extracts were filtered with medium-grade filter paper, lyophilized and stored at −80°C. Stock solutions were prepared by dissolving 163.8 mg of the freeze-dried extract in 1 mL DMSO.

### 1.3 HPLC analysis of *A. camphorata* extracts

The quantitative study of three standard compounds (AC-1, AC-2, AC-3) in *A. camphorata* extracts were performed by HPLC. The HPLC system consisted of a reversed-phase column on an Alliance HPLC (Waters, e2695 Separations modules) equipped with an autosampling and controller with dual pump, a 2998 photodiode array detector (PDA) and Empower software. Each 20 µL sample solution was applied to the analytic column. Separation was performed in a Merck (50995) LiChrospher RP-18 column (4.6 mm×250 mm, 5 µm), at a flow rate of 1 mL/min, and the absorbance was detected at 245 nm. The mobile phase consisted of A (water) and B (100% acetonitrile): 0–90 min, 100–0% A and 0–100% B.

### 1.4 Bacterial strains and growth conditions

Reference bacterial strains representative of oral microorganisms included *P. gingivalis* BCRC 14417 (ATCC 33277) and *S. mutans* BCRC 15256 (ATCC 31383). Brain-heart infusion (BHI) broth was used for the growth of *S. mutans*. For the growth of *P. gingivalis*, BHI broth supplemented with yeast extract (5 mg/mL) and cysteine hydrochloride (0.05%), hemin (5 µg/mL) and vitamin K1 (1 µg/mL) was used. Bacteria were cultured anaerobically (85% N2, 10% H2, and 5% CO2) at 37°C.

### 1.5 Minimal inhibitory concentration

Antibacterial assays of *A. camphorata* extracts and three standard compounds (AC-1, AC-2, AC-3) were performed according to the broth microdilution method. Cultures of the bacteria were added to culture medium containing a series of dilutions of extracts of *A. camphorata* in the wells of microtiter plates, and bacterial growth was assessed after an incubation period. The inoculum size was controlled by measuring the optical density at 600 nm. Successive two-fold dilutions of *A. camphorata* extracts were prepared in a 5 µL volume, and 195 µL of the bacterial culture was added to the prepared plates. The final inoculum concentration of the periodontopathic bacteria (*P. gingivalis*) or cariogenic bacteria (*S. mutans*) was 1×10^5^ CFU/mL. The plates included wells containing one growth control and one sterile control. Chlorhexidine was used as a positive control. The final concentration of extracts and the positive control ranged from 2048 µg/mL to 1 µg/mL. After incubation under anaerobic conditions at 37°C for 24 hours, the level of microbial growth was measured with a microplate reader at 600 nm. The minimal inhibitory concentration (MIC) was defined as the lowest dilution of *A. camphorata* extract that restricted growth to an absorbance of ≤0.1.

### 1.6 Minimal bactericidal concentration

The minimal bactericidal concentration (MBC) was determined in 96 well microplates with 195 µL of inoculum (1×10^5^ CFU/mL) in BHI broth and 5 µL of extract or control. For the growth of *S. mutans*, the microplaque was incubated for 24 hours at 37°C in 15% CO_2_. An aliquot of all incubated test wells was subcultured on BHI agar, and all plates were incubated at 37°C in an anaerobic jar with a gas generating kit for 1 day.

For the growth of *P. gingivalis*, vials with an inoculum of approximately 10^5^ CFU/mL in supplemented BHI broth and containing increasing concentrations of each extract of *A. camphorata* (2∼2048 µg/mL) and three standard compounds (AC-1, AC-2, AC-3) were incubated for 48 hours. After duplication, 0.1 mL of the diluted cultures was inoculated onto the surface of BHI agar supplemented with 5% defibrinated sheep blood. Plates were incubated at 37°C in anaerobiosis for 5 days. For the growth of *S. mutans* and *P. gingivalis*, the minimal bactericidal concentration was determined as the A. camphorata extract concentration that killed 100% of the bacterial inoculum. All assays were performed in duplicate.

### 1.7 Adherence inhibition assay of *S. mutans*

Adherence inhibition assays were done using the protocol of Kang et al. (21). Briefly, BHI broth containing 2% sucrose and various concentrations of each extract of *A. camphorata* dissolved in dimethylsulfoxide (DMSO) to prepare test reagents from 2 to 64 µg/mL and then mixture was inoculated with overnight cultures of *S. mutans*. Blank controls consisted of cells grown in BHI broth with sucrose only and chlorhexidine which is a clinically proven anti-plaque agent was used as a positive control. Media and non-adherent bacterial cells were decanted from the wells, and the remaining loosely bound cells were removed by rinsing twice with distilled water. The plates were then blotted on paper towels and air dried, and adherent bacteria were stained with 50 µL of 0.1% crystal violet for 15 min at room temperature. After rinsing twice with 200 µL of distilled water, the bound dye was extracted from the stained cells with 200 µL of 99% ethanol. The adherent bacteria were measured with a microplate reader at 570 nm.

### 1.8 Cytotoxicity of *A. camphorata* extracts on HGF cells

Normal human gingival fibroblast (HGF) cells were grown in DMEM supplemented with 4.5 g/L glucose, 2 mM *L*-glutamine (Gibco, USA), 1% penicillin-streptomycin (Gibco, USA) and 10% fetal bovine serum (Gibco, USA) in 5% CO_2_ at 37°C. The cytotoxic effects of the different *A. camphorata* extracts on HGF cells were measured using an MTT assay. The cells were seeded at a density of 5×10^3^ cells per well in 96-well culture plates overnight and then treated with various concentrations (8∼512 µg/ml) of the extracts. After 24 hours of incubation, culture medium containing MTT (1 mg/ml) was added to each well, and the plate was incubated for another 2 hours. Consecutively, the medium was removed, and DMSO was added to extract the MTT formazan. The absorbance of each well was measured with an enzyme-linked immunosorbent assay (ELISA) reader at 570 nm.

### 1.9 Statistical analysis

All values are presented as means±SE.; the comparison of means was performed with the SPSS for Windows statistical software package (SPSS Inc., USA). Differences were considered significant at P<0.05.

## Results

### HPLC determination of *A. camphorata* extracts

**Figure 1:**
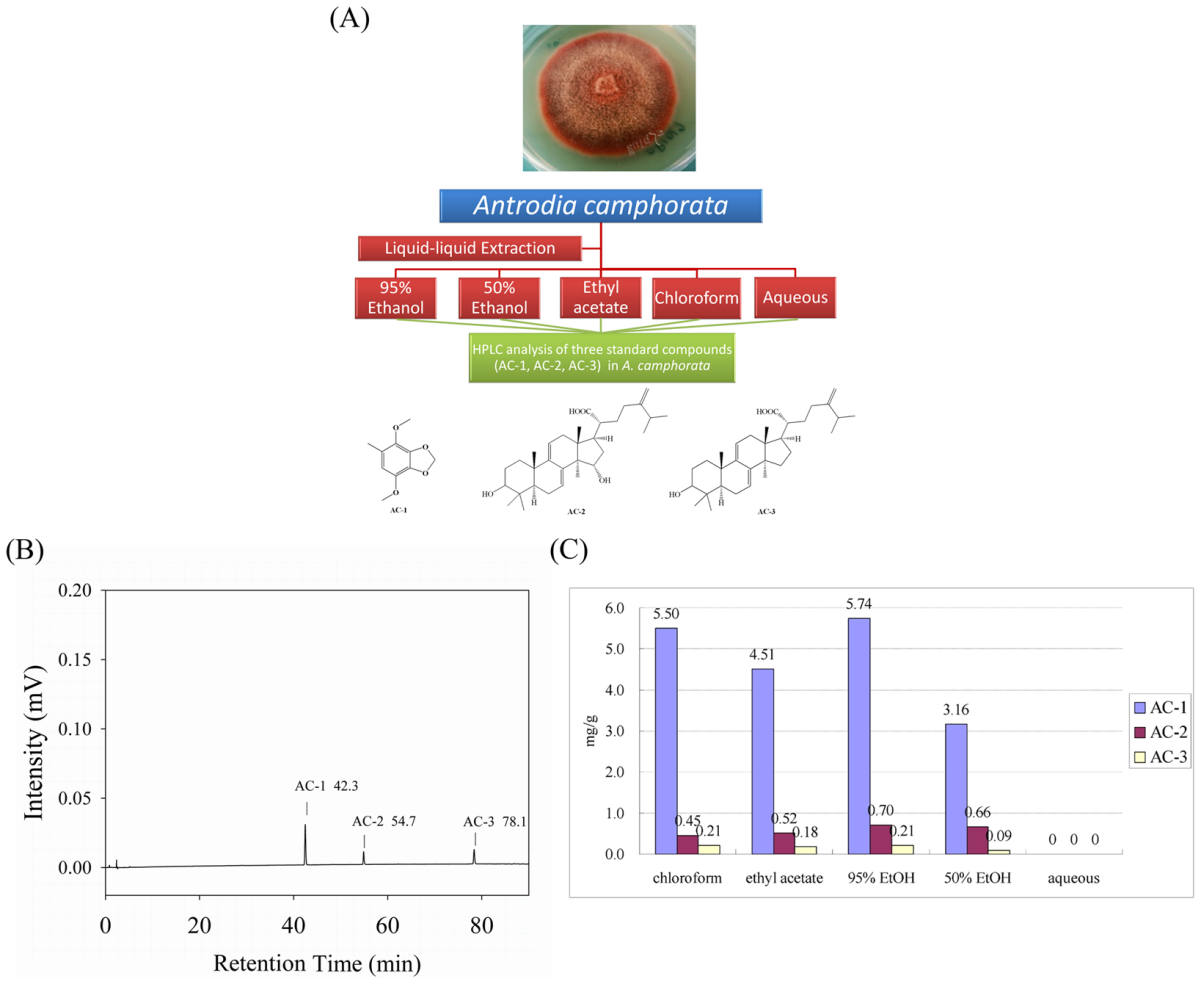
Diagram of separation and chemical analysis of *A. camphorata* extracts. (A) A schematic representation of a liquid-liquid extraction and bioassay process for *A. camphorata*. (B) HPLC chromatogram of three reference standards of *A. camphorata* with corresponding retention times at λ = 245 nm, 4,7-dimethoxy-5-methyl- l,3-benzodioxole (AC-1) (retention time = 42.3 min); dehydrosulphurenic acid (AC-2) (retention time = 54.7 min); dehydroeburicoic acid (AC-3) (retention time = 78.1 min). Separation was performed in a Merck (50995) LiChrospher RP-18 column (4.6 mm×250 mm, 5 µm), at a flow rate of 1 mL/min. The mobile phase consisted of A (water) and B (100% acetonitrile): 0–90 min, 100–0% A and 0–100% B.(C) Quantitative analysis of three standards in five *A. camphorate* extracts: chloroform (CHCl_3_), ethyl acetate (EA), 95% ethanol (EtOH), 50% ethanol (EtOH), and H_2_O.

Dried *A. camphorata* was used to prepare various extracts with five different polarity solvents such as water, 50% ethanol, 95% ethanol, ethyl acetate and chloroform, and moreover three bioactive components AC-1, AC-2, AC-3 in those crude extracts have been analyzed by gradient high-performance liquid chromatography. Qualification and quantification analysis was accomplished on an Alliance HPLC (Waters, e2695 Separations modules) equipped with a Merck (50995) LiChrospher RP-18 column (4.6 mm×250 mm, 5 µm), at a flow rate of 1 mL/min, and the absorbance was detected at 245 nm as shown in Figure 1B.

The presence and quantity of three reference chemicals within *A. camphorata* were confirmed as shown in Figure 1C. Four crude extracts from organic solvents consisted component AC-1, AC-2 and AC-3 except the aqueous extract which was not detected any of those three standards, whereas the 95% ethanol, ethyl acetate and chloroform extracts contained similar concentrations of all compounds tested.

### Antibacterial activity of *A. camphorata* extracts against oral bacteria

**Figure 2:**
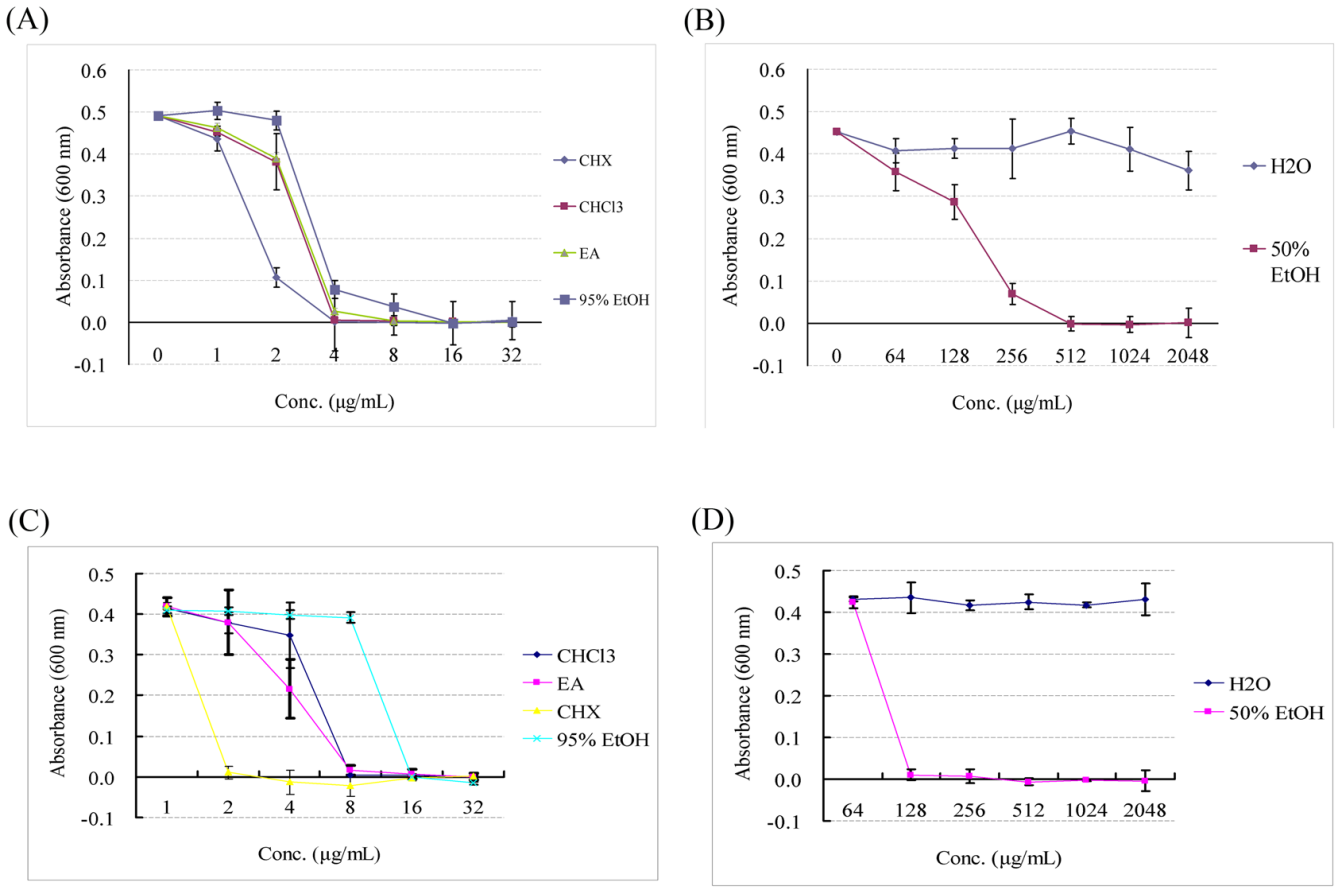
Determination of the antibacterial activity of *A. amphorata* extracts and chlorhexidine against *P. gingivalis* (Figure 2A and Figure 2B) and *S. mutans* (Figure 2C and Figure 2D). Serial doses of extracts were added to the bacterial cultures in 96-well plates and incubated for 24 hours. Bacterial growth was determined by measuring the optical density of the cultures at 600 nm. The data are reported as the means±SD of triplicate wells. *P<0.05.

The antibacterial activity of *A. camphorata* extracts against *P. gingivalis* and *S. mutans* was determined with a broth microdilution method. The MIC results for the aqueous, ethanol, ethyl acetate and chloroform extracts of *A. camphorata* are shown in Table 1. Among the five extracts, the 95% ethanol, ethyl acetate and chloroform extracts exhibited the lowest MIC against *P. gingivalis* and *S. mutans* (MIC = 4∼16 µg/mL, Fig 2A and Fig 2C), followed in ascending order by the 50% aqueous-ethanol extract (MIC = 128 µg/mL, Fig 2B and Fig 2D). As a positive control, chlorhexidine had strong antimicrobial activity against periodontopathic bacteria (MIC = 2∼4 µg/mL, Fig 2A and Fig 2C). The MIC of the aqueous extract was greater than 2048 µg/mL against both *P. gingivalis* and *S. mutans*.

**Table 1:** MIC and MBC of *A. camphorata* extracts against strains of representative oral pathogens by the micro-dilution method.

Extracts	MIC			MBC			
	µg/mL			µg/mL			
	*S. mutans*	*P. gingivalis*	Range	*S. mutans*	Range	*P. gingivalis*	Range
Chloroform	8	4	1–32	64	16–1024	4	2–32
Ethyl acetate	8	4	1–32	32	16–1024	4	2–32
95% Ethanol	16	4	1–32	64	16–1024	8	2–32
50% Ethanol	128	128	64–2048	1024	32–2048	256	32–2048
Water	>2048	>2048	64–2048	>2048	32–2048	>2048	32–2048
Chlorhexidine	2	4	1–32	16	2–32	4	2–32

The MBCs for the *A. camphorata* extracts are also presented in Table 1; the ethanol, ethyl acetate and chloroform extracts exhibited bactericidal activity. The lowest MBCs against *P. gingivalis* and *S. mutans* (4∼16 µg/mL) were exhibited by the ethanol, ethyl acetate and chloroform extracts. The MBC values of *P. gingivalis* and *S. mutans* treated with the 50% aqueous-ethanol extract were 256 and 1024 µg/mL, respectively. *P. gingivalis* and *S. mutans* exhibited low sensitivity to the aqueous extract (MBC>2048 µg/mL). Chlorhexidine exhibited a lower inhibitory effect on the growth of both bacteria, with an MBC of 4 µg/mL for *P. gingivalis* and 2 µg/mL for *S. mutans*.

In this study, we firstly investigated the inhibitory effects of three standard compounds AC-1, AC-2, AC-3 which are important bioactive components in *A. camphorata*. The cells were treated with various concentrations of compounds AC-1, AC-2, AC-3 as shown in Table 2 and *P. gingivalis* was significantly suppressed by compound AC-1. The ethanol, ethyl acetate and chloroform extracts of *A. camphorata* contained the highest concentrations of the three standards especially compound AC-1 and also exhibited more potent bactericidal activity against the growth of *P. gingivalis* comparable to that of the aqueous extract.

**Table 2:** MIC and MBC of the three standard compounds against *P. gingivalis*.

Compound	MIC	Range	MBC	Range
	µg/mL		µg/mL	
AC-1	128	32–1024	512	64–1024
AC-2	>1024	32–1024	>1024	64–1024
AC-3	>1024	32–1024	>1024	64–1024
Chlorhexidine	2	1–32	4	2–32

### Inhibitory effects of *A. camphorata* extracts on *in vitro* adherence of *S. mutans*

As shown in Table 3, *in vitro* adherence of *S. mutans* was significantly inhibited by the addition of the ethyl acetate and chloroform extracts (16∼24 µg/mL) and ethanol extract (32∼64 µg/mL) compared with the control (P<0.05). The chloroform extract (16 µg/mL) and ethanol extract (32 µg/mL) yielded greater than 50% inhibition. Both the ethyl acetate and chloroform extracts of *A. camphorata* inhibited the adherence of cariogenic bacteria at low doses.

**Table 3:** Inhibitory effects of *A. camphorata* extracts on *S. mutans in vitro* adherence.

Concentration (µg/mL)	Adherence inhibition of *S. mutans* (% of control)			
	*A. camphorata extracts*			Positive control
	*Ethanol*	Ethyl acetate	Chloroform	Chlorhexidine
0 (control)	0.0%±0.0%	0.0%±0.0%	0.0%±0.0%	0.0%±0.0%
2	−0.1%±1.7%	3.4%±3.1%	5.1%±3.9%	−0.2%±1.2%
4	0.1%±0.8%	0.3%±0.3%	0.4%±0.3%	28.9%±4.1%*
8	−1.0%±0.4%	2.6%±6.4%	1.1%±0.6%	79.7%±4.6%*
16	0.5%±1.2%	50.9%±3.9%*	53.0%±3.0%*	93.4%±0.7%*
32	79.7%±4.7%*	94.4%±0.7%*	93.3%±0.8%*	95.4%±0.3%*
64	95.4%±0.3%*	95.9%±1.5%*	96.9%±0.5%*	96.9%±0.5%*

Data are expressed at Mean±SD of representative experiment performed in triplicate.

*P<0.05 as compared with control.

### Cytotoxicity

**Figure 3:**
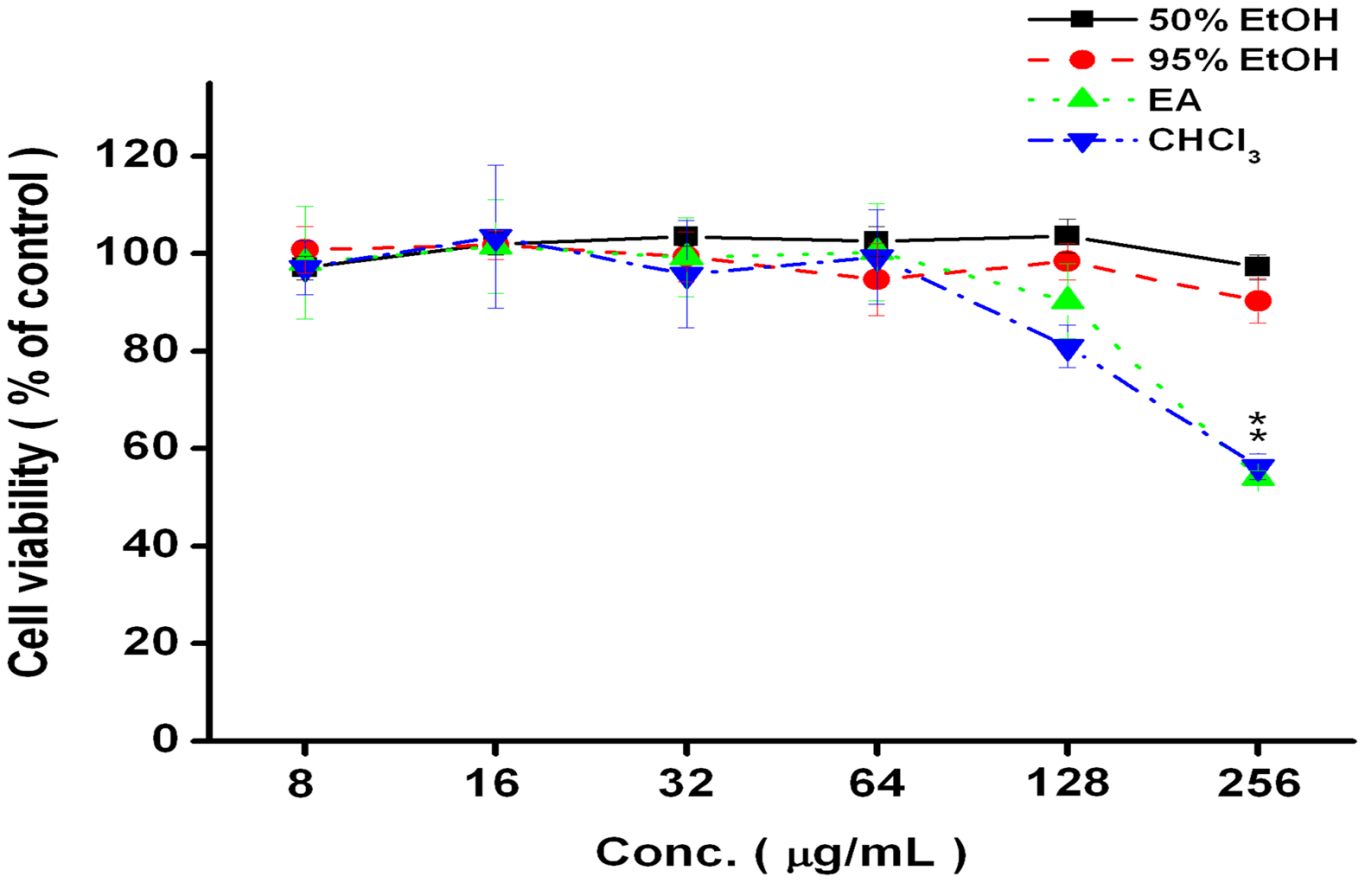
Cytotoxicity of different extracts (50% ethanol (EtOH), 95% ethanol (EtOH), ethyl acetate (EA) or chloroform (CHCl_3_) of *A. camphorata* against HGF cells. HGF cells were treated with extracts at various doses for 24 hours. Untreated cells were used as a control. Cell viability was assessed by an MTT assay. Six samples were analyzed in each group. The values represent the means±SE.* means significant between treatment and control. (P<0.001).

Cell viability was measured using an MTT assay. The viabilities of HGF cells incubated with the 50% ethanol extract at concentrations of 8, 16, 32, 64, 128 and 256 µg/ml for 24 hours were 97.1±2.4%, 102.0±2.3%, 103.5±1.9%, 102.5±3.0%, 103.6±3.5% and 97.2±2.6% of the control value, respectively. The viabilities of HGF cells incubated with the 95% ethanol extract (8∼256 µg/ml) for 24 hours were 100.8±4.8%, 101.8±3.0%, 99.4±5.1%, 94.7±7.4%, 98.4±3.8% and 90.3±4.5% of the control value, respectively. The viabilities of HGF cells incubated with the ethyl acetate extract (8∼256 µg/ml) for 24 hours were 98.1±11.6%, 101.5±9.7%, 99.2±8.1%, 100.3±10.0%, 90.3±7.7% and 54.0±1.4% of the control value, respectively. The viabilities of HGF cells incubated with the chloroform extract (8∼256 µg/ml) for 24 hours were 97.1±5.6%, 103.5±14.6%, 95.7±11.0%, 99.3.±9.7%, 80.9±4.4% and 56.3±5.7% of the control value, respectively. No obvious cytotoxic effects of the 50% and 95% ethanol extracts were observed. However, the ethyl acetate and chloroform extracts (256 µg/ml) significantly decreased cell viability (as shown in Figure 3).

## Discussion

*S. mutans* is a paradigm for the virulence of dental caries, and the main virulence factors of *S. mutans* associated with cariogenicity include adhesion, acidogenicity, and acid tolerance. The ability of *S. mutans* to synthesize glucans from sucrose increases the efficiency of adhesion and enhances the proportion of *S. mutans* within dental plaque, and *S. mutans* adherence plays a significant role in initiating the changes in plaque ecology that can lead to dental caries. In our study, *in vitro* adherence of *S. mutans* was significantly inhibited by the ethyl acetate and chloroform extracts (16∼24 µg/mL), while the ethanol extract (32∼64 µg/mL) exhibited moderate inhibitory activity. Whereas the antibacterial activity of the 95% ethanol, ethyl acetate and chloroform extracts of *A. camphorata* against *S. mutans* were weaker than that of chlorhexidine, as shown in Figure 1(C).

Plaque-induced inflammatory lesions are associated with periodontal diseases, and collagen degradation is observed in chronic periodontal disease. *P. gingivalis* has also been linked to rheumatoid arthritis [17]. Thus, effective antimicrobial agents against *S. mutans* and *P. gingivalis* have been the subject of research and development. A common antiseptic mouthwash agent, chlorhexidine, presumably reduces populations of *S. mutans* by interfering with bacterial adherence, but it may have some side effects including skin irritation, itching, or redness. Other antimicrobial agents, such as fluoride, xylitol, green tea extract, and tea tree oil, are also used in dentistry. In addition, herbal extracts and components derived from *Magnoliae cortex* have been demonstrated to have antimicrobial activity against *S. mutans* [18] and *P. gingivalis* [19]. Anti-bacterial agents from natural herbal extracts, such as methyl gallate and gallic acid, the main compounds of gallotannins in *Galla Rhois*, exhibit significant inhibitory activity against cariogenic and periodontopathic pathogens and the formation of *S. mutans* biofilms [20]–[21]. Moreover, EGCg, which is found in green tea, is a natural anti-cariogenic agent that exhibits antimicrobial activity against *S. mutans* and suppresses the specific virulence factors associated with its cariogenicity [22].

In our study, various *A. camphorata* extracts exhibited inhibitory activity; in particular, the 95% ethanol, ethyl acetate and chloroform extracts of *A. camphorata* exhibited potent bactericidal activity against both *S. mutans* and *P. gingivalis*. In general, the concentration of the extracted aliphatic compounds decreased when the water content of the extraction solvent increased. Aliphatic compounds have previously been reported as effective antimicrobial agents against oral pathogens. For example, methyl gallate may be safer and more effective than gallic acid to be a better candidate for treating dental caries and periodontal diseases [21]. Regarding the polarity of the compositions of the *A. camphorata* extracts prepared in different solvents, the antimicrobial activity and safety of the ethanol extract may be most appropriate for oral health and hygiene. Interestingly, a triterpenoid, methyl antcinate A (MAA), which was isolated from *A. camphorata*, exhibits a potent spectrum of anticancer effects in four different oral cancer cell lines (TSCCa, GNM, OC-2, and OEC-M1) [23].

Compound AC-1 significantly suppressed the growth of *P. gingivalis*, whereas neither compound AC-2 nor compound AC-3 showed significant inhibitory effects against *P. gingivalis*. Bacteria growth inhibitory effects of the crude extracts of *A. camphorata* exhibited more potent than compound AC-1, so it may be indicating that independently these reference chemicals are not major responsible for the MIC and MBC activities exhibited by the extracts in which they are present. Although compound AC-1 has been clearly contributed to the activity exhibited by the extracts, however the other components not tested or combinations of the chemicals within *A. camphorata* may be also contributing to the overall inhibitory activities against oral bacteria. Nevertheless, *A. camphorata* extracts indeed may be a beneficial and effective approach for preventing oral diseases and could be incorporated into solutions to help patients maintain a daily oral hygiene regimen and control caries and periodontal disease in the future.

## Conclusions

In conclusion, an ethanol extract of *A. camphorata* can inhibit the proliferation of *S. mutans* and *P. gingivalis* and the *in vitro* adherence of *S. mutans*, but has no cytotoxicity to HGF cells. The inhibition is likely to reduce the virulence of these cariogenic and periodontopathic bacteria and reduce the rate of dental plaque formation. The use of *A. camphorata* extracts may be safer and more effective for controlling oral pathogens and diseases.
